# A dataset of global tropical cyclone wind and surface wave measurements from buoy and satellite platforms

**DOI:** 10.1038/s41597-024-02955-4

**Published:** 2024-01-22

**Authors:** Ali Tamizi, Ian R. Young

**Affiliations:** Department of Infrastructure Engineering, University of Melbourne, Melbourne, Australia

**Keywords:** Physical oceanography, Civil engineering

## Abstract

There are now a range of potential data sources for wind and surface wave conditions within tropical cyclones. These sources include: *in situ* buoy data and remote sensing data from satellite altimeters, scatterometers, and radiometers. In addition, data providing estimates of tropical cyclone tracks and wind field parameters are available from best track archives. The present dataset brings together this information in a single archive, providing the available data for each tropical cyclone from each of the data sources in a single file. The data consists of observations in a total of 2927 global tropical cyclones over the period from 1985 to 2017. Global statistics of the observations are provided, along with data on the geographic distribution of tropical cyclones within the database.

## Background & Summary

In tropical and subtropical regions, tropical cyclones (TCs; hurricanes or typhoons) represent the major extreme meteorological events. The strong winds and extreme waves associated with such systems are of critical importance for a range of applications and processes. These include: coastal and offshore engineering design, coastal beach erosion and coastal inundation, the safety of shipping and studies of extreme air-sea interaction. The extreme winds and complex vortex structure of the moving tropical cyclone, are also a significant test for our understanding of wind-wave generation and propagation. As such, tropical cyclones are often regarded as one of the most demanding tests of contemporary wind-wave prediction models.

Despite the importance of tropical cyclones, obtaining consistent, reliable, and extensive datasets of wind and wave conditions in such systems is demanding. The relatively small geographic extent of TCs, their infrequent occurrence and the extreme nature of the meteorological forcing, mean observations are relatively rare. The most obvious source of observations of TC wave fields is the use of *in situ* buoy data, which has been the subject of numerous studies^1–11^. These studies have largely used the U.S. National Data Buoy Center (NDBC) dataset^12^, as it is by far the most comprehensive archive. Buoys provide high quality data, which have been extensively validated and these data often consist of full directional spectra. The limited spatial distribution of buoys, however, which are often relatively close to coastlines, limits the data available.

The advent of remote sensing techniques has significantly increased the potential available data, with both Synthetic Aperture Radar (SAR) and altimeters having been utilized to observe TC wave fields^11,13–21^.

Observations of the TC wind field over the ocean have similarly relied upon buoy measurements. However, the accuracy of such measurements is sometimes questioned due to tilting of the anemometer on the buoy and sheltering by waves^11,22–24^. The routine penetration of North American hurricanes by aircraft has provided the opportunity to obtain TC wind field data from dropwindsondes^25–29^. As such measurements are, however, aircraft-based, they are obviously limited in terms of the number of TCs penetrated. As with wave measurements, the advent of remote sensing measurements has expanded the combined wind field database. These systems include aircraft-borne Stepped Frequency Microwave Radiometers (SFMR)^30,31^. Satellite-based instruments include: the Advanced Microwave Sounding Unit (AMSU)^32^, the L-band microwave radiometer carried on the NASA Soil Moisture Active Passive Satellite (SMAP)^33^, the CYGNSS constellation^34^, and scatterometers^35–38^.

It should be noted that none of these data sources are homogeneous. The numbers of buoys deployed and satellites in orbit have increased with time, meaning that the observation frequency of TCs has changed over the last 30 years. In addition, the measurement technology has also changed. In recent years more buoys measure full directional spectra, rather than just bulk parameters, such as significant wave height and peak period. Similarly, the satellite technology has also changed with higher resolution and more consistent calibration of platforms.

In a series of papers, Tamizi and Young^11^ and Tamizi *et al*.^38,39^ combined data from the TC best track database IBTrACS^40^ with buoy, altimeter, scatterometer and radiometer data to provide a large and comprehensive investigation of the wind and wave fields in TCs. In these studies, TC tracks were identified globally from the IBTrACS archive. NDBC buoy data and the various remote sensing products were then extracted when the given TC track passed close (550 km or 5°) to the buoy or satellite ground track. This is a large dataset, encompassing a total of 2927 TCs from all tropical cyclone basins. As data is sourced from a range of public archives, which each need to be separately searched to extract the required observations, this is not a simple task. The present database contains these data, stored by year/location/TC name. As such, it is a valuable archive bringing together TC track and wind field parameters with recorded wind and wave data.

## Methods

Each of the data types used in the composite dataset are described below. Note that in the previous applications of these data^11,38,39^, track positions were interpolated in time to ensure they were consistent with observations of wave and wind quantities. The present dataset does not interpolate any data. Rather, the original track observations are provided at their original resolution (6 hours in most cases).

### Tropical cyclone tracks and parameters (IBTrACS)

The International Best Track Archive for Climate Stewardship (IBTrACS) dataset (40) was developed by the NOAA (National Oceanographic and Atmospheric Administration) National Climatic Data Center. The archive synthesizes and merges best-track data from all official Tropical Cyclone Warning Centers and the WMO (World Meteorological Organisation) Regional Specialized Meteorological Centers. The dataset contains data including time, position, maximum sustained winds, minimum central pressure, *p*_0_ and storm nature (i.e., tropical cyclone, tropical storm, etc.). In addition, information such as the radius to maximum winds, *R*_*m*_ and the radius to gales *R*_34_ is provided for some storms. The data are provided globally at 6-h intervals. Although the archive contains data beginning from 1848, data before the satellite period is obviously of lower quality. For the present database, only data after 1985 was extracted.

**Fig. 1:**
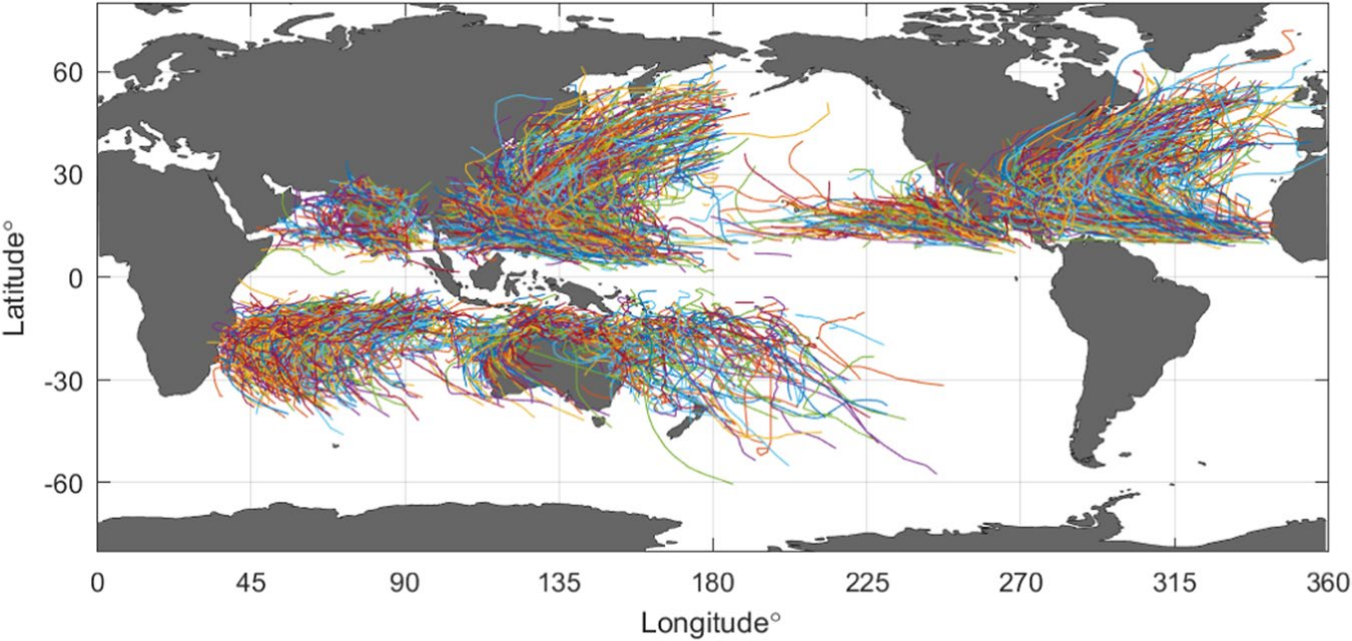
Tracks of tropical cyclones extracted from IBTrACS^60^ and contained within the database. For clarity, only every 4^th^ track is shown. (Figure created with Matlab R2023a –mathworks.com).

Figure 1 shows the global distribution of tropical cyclone tracks extracted from IBTrACS and contained within the dataset. The tracks, as shown in Fig. 1 contain data for storms which are classified as tropical storms, tropical cyclones, and extra tropical cyclones. The present database includes all storm types. If one wished to exclude extratropical cases, as in Tamizi and Young^11^, one can simply disregard data at latitudes higher than 40°.

### NDBC Buoy data

The NDBC operates the longest duration deep water wave buoy network in the world^12^. Of particular relevance for the present application, this network covers the Atlantic, Pacific, and Gulf of Mexico regions where North American hurricanes occur. The NDBC buoy data typically includes hourly measurements of significant wave height, *H*_*s*_ and other bulk parameters (wave period etc), and the one-dimensional energy density spectrum *E*(*f*), where *f* is wave frequency. In addition, for directional buoys, NDBC data contains the cross-spectral moments *a*_1_, *a*_2_, *b*_1_ and *b*_2_. The significant wave height can be related to *E*(*f*) and the directional energy density spectrum, *E*(*f, θ*), where *θ* is wave propagation direction, by

1 $${H}_{s}^{2}/16=\int E(f,\theta )df\,d\theta =\int E(f)df$$

The directional wave spectrum, *E*(*f, θ*) can be represented as *E*(*f, θ*) = *E*(*f*)*D*(*f, θ*)^41,42^, where *D*(*f, θ*) is a directional spreading function defined such that $\int D(f,\theta )d\theta =1$. In an approach termed the Fourier Expansion Method (FEM), Longuet-Higgins *et al*.^41^ proposed that *D*(*f, θ*) takes the form

2 $$D(f,\theta )=A{(f)\cos }^{2s(f)}\left[\frac{\theta -{\theta }_{m}(f)}{2}\right]$$

The mean direction *θ*_*m*_(*f*) and the spreading parameter *s*(*f*) can be determined from the first two spectral moments *a*_1_(*f*) and *b*_1_(*f*) as

3 $${\theta }_{m}(f){=\tan }^{-1}\left[{b}_{1}(f)/{a}_{1}(f)\right]$$

4 $$s(f)=\frac{{r}_{1}(f)}{1-{r}_{1}(f)}$$

where ${r}_{1}^{2}(f)={a}_{1}^{2}(f)+{b}_{1}^{2}(f)$. The coefficient *A*(*f*) is a normalization factor. Although the FEM defined by (2) to (4) is a useful representation of the directional spectrum, the assumed cos^2*s*^ form is a significant simplification. More general representations such as the Maximum Likelihood Method^42,43^ and Maximum Entropy Method^44^ can also be defined in terms of the cross-spectral moments *a*_1_, *a*_2_, *b*_1_, *b*_2_.

The NDBC buoys also measure wind speed. As anemometers on these buoys are at a range

of heights, measurements need to be converted to a standard reference height, usually 10 m, assuming a neutral stability logarithmic boundary layer^45,46^.

**Fig. 2:**
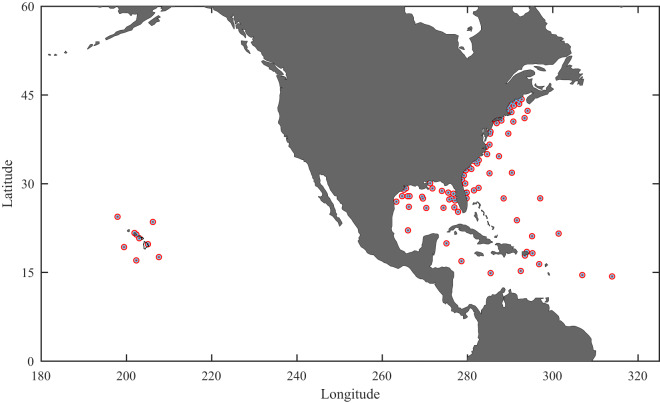
Locations of NDBC buoys for which tropical cyclone *in situ* wind speed and wave data are included in the database. (Figure created with Matlab R2023a – mathworks.com).

The locations of the NDBC buoys from which TC data are included in the database are shown in Fig. 2. Note that tropical cyclone (hurricane) wave data are also available from the Coastal Data Information Program (CDIP). However, as these data are generally in finite depth locations, it was not included in the present database, which is for deep water.

The buoy data for the present database were downloaded from the NDBC^47^ archive (https://www.ndbc.noaa.gov/). The same data can also be accessed in a more accessible form as described by Hall and Jensen^48^. In the present archive, the quantities, *r*_1_, *r*_2_ and *θ*_*m*_ were calculated as above. All other quantities are as in the NDBC archive.

### Altimeter data

Radar altimeters have been in operation since 1985 and measure wind speed (*U*_10_) and significant wave height (*H*_*s*_) globally at an along-track resolution of approximately 10 km. As the radar altimeter is a “nadir-looking” instrument, it senses the ocean surface over a narrow beam directly below the satellite. As such, the cross-track resolution is low, with ground tracks separated by hundreds of kilometres. As the geographical extent of TCs is relatively small, altimeter data are not always available for such meteorological systems. Due to the global coverage and temporal extent (1985 to present-day) of the combined altimeter missions, however, there are extensive observations of TC wind and wave fields. Ribal and Young^45^, have developed a consistent database of global altimeter data from the 13 altimeters which were operational from 1985–2018. This database^49^ has now been extended to 15 altimeters and is available at https://portal.aodn.org.au/. Although there is some degradation of altimeter data in high rain regions, a large quality-controlled dataset is available under TC conditions^11^.

The altimeter data base has been calibrated against buoy data and cross-validated at cross-over points between altimeter missions operational at the same time^45^.

### Scatterometer data

Scatterometers have been in operation globally since 1992 and measure wind speed (*U*_10_) and direction (*θ*_*u*_) at a resolution of between 12.5 km and 25 km. In contrast to altimeters, scatterometers measure over a broad swath up to 1400 km wide. As such, they image most locations on the Earth’s surface twice per day. Ribal and Young^50^ calibrated the various scatterometer missions since 1992 against buoy data. However, these calibrations are limited to wind speeds up to approximately 30 ms^−1^. At higher wind speeds, scatterometers display a low bias due to reduced backscatter signal^51,52^. Chou *et al*.^53^ have proposed a correction to ASCAT scatterometer winds to address this issue at high winds. This approach was extended by Ribal *et al*.^54^ who developed specific correction relationships for the MetOp-A, MetOp-B, ERS-2 and OceanSat-2 scatterometers. For QuikSCAT they found no correction is required. These corrections can be applied to the data of Ribal and Young^45^ for application in TC conditions. The present database uses the calibrations of Ribal and Young^45^.

**Fig. 3:**
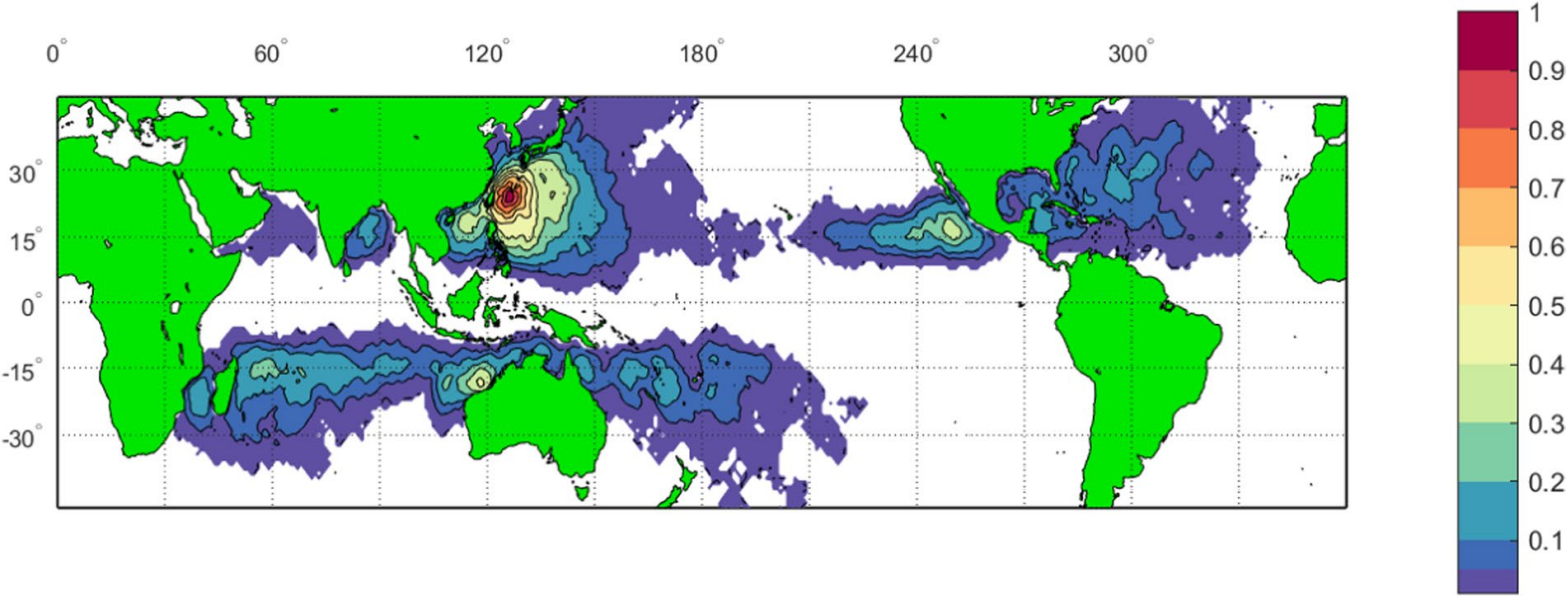
Contour plot of the relative density (maximum value 1.0) of scatterometer observations within the TC database. (Figure created with Matlab R2023a – mathworks.com).

Figure 3 shows a contour plot of the relative density of scatterometer observations of winds within the present TC database. As one would expect, it closely follows the distribution of TC tracks shown in Fig. 1.

#### Radiometer data

In a similar fashion to scatterometer, radiometers measure over a broad ground track swath with a spatial resolution of 25 km. The radiometer dataset^55^ is extensive, commencing in 1986. However, radiometer returns are significantly degraded by heavy rain. As such, radiometer data can generally only provide reliable data in TC conditions for the periphery of storms.

### Composite data records for tropical cyclones

**Fig. 4:**
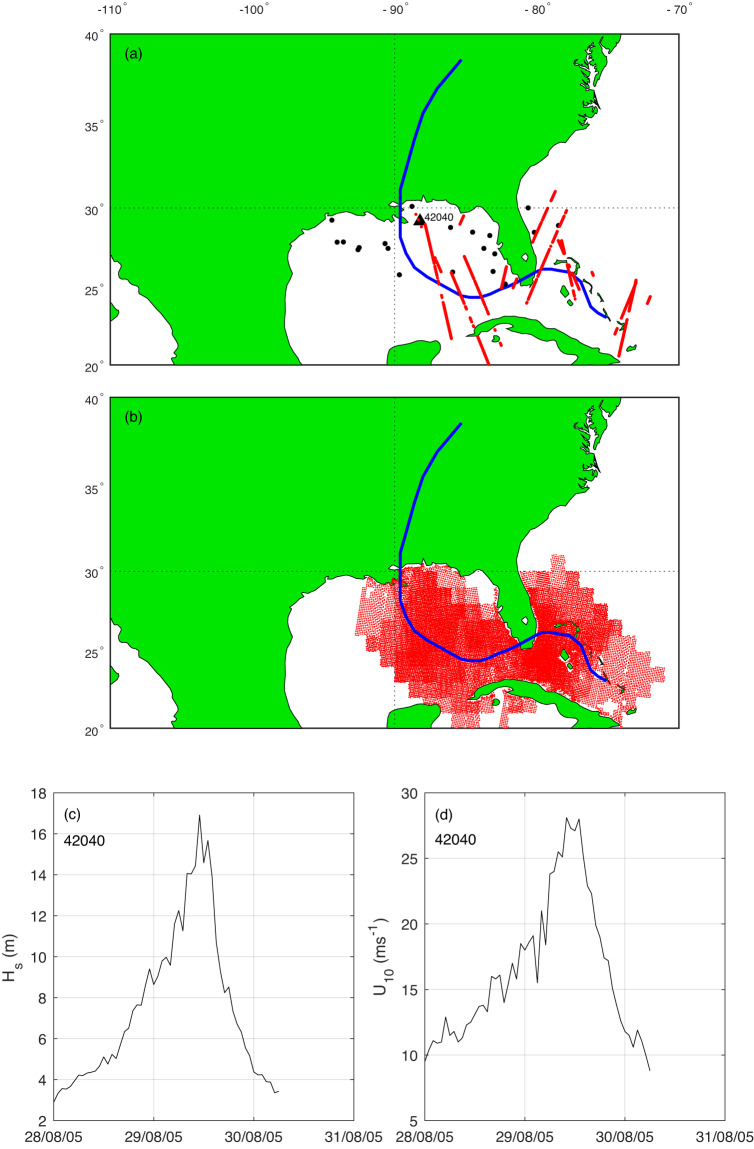
Observations of wind and wave conditions during Hurricane Katrina during 2005. (**a**) TC track in blue, buoy locations shown with red dots and altimeter observations by linear tracks. The location of NDBC Buoy 42040 is shown. (**b**) ground track swaths of scatterometer wind data. (**c**) significant wave height (*H*_*s*_) as a function of time from buoy 42040. (**d**) wind speed (*U*_10_) as a function of time from buoy 42040. (Figure created with Matlab R2023a – mathworks.com).

As noted above, these combined datasets provide detailed observations of wind and wave properties under TC conditions. A typical example of the composite data is shown in Fig. 4, with the track and available data for buoys, altimeter and scatterometer for Hurricane Katrina in the Caribbean and Gulf of Mexico during 2005. The data were extracted from database file 2005236N23285_KATRINA.nc. The figure shows the broad distribution of buoy data available from the NDBC archive, the extensive altimeter tracks across the track of the hurricane and the broad swaths of scatterometer data. The *in situ* data at Buoy 42040 shows *H*_*s*_ peaking at approximately 17 m and *U*_10_ at 28 ms^−1^.

**Fig. 5:**
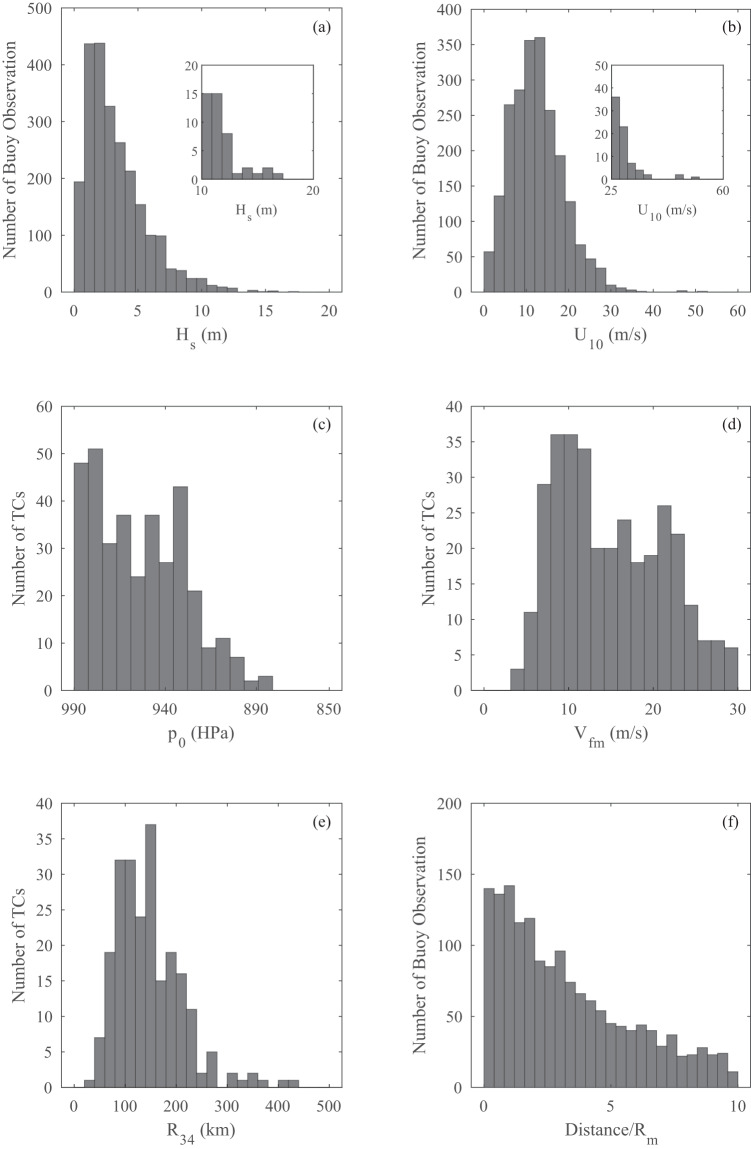
Summary of data from the in situ buoy TC database: (**a**) *H*_*s*_ for each transect of a TC, (**b**) *U*_10_ for each transect, (**c**) *p*_0_ of each storm in the database, (**d**) *V*_*fm*_ of each storm in the database, (**e**) *R*_34_ of each storm in the database, and (**f**) minimum distance between TC eye and buoy for each case in the database.

Figure 5 shows histograms of wind, wave and TC parameters for data associated with the buoy observations in the database. More details are provided in Tamizi and Young^11^. The database of buoy observations consists of more than 2900 time series of wind speed/wave height from more than 350 TCs. The histograms show *H*_*s*_ up to 18 m and *U*_10_ up to 60 ms^−1^. These values were recorded during the passage of TCs (hurricanes) with central pressure, *p*_0_ down to 880HPa and values of velocity of forward movement, *V*_*fm*_ up to 30 ms^−1^. The data were taken within 10 times the radius to maximum wind, *R*_*m*_ of the TC centre, with the TCs having values of radius to gales, *R*_34_ up to 420 km.

**Fig. 6:**
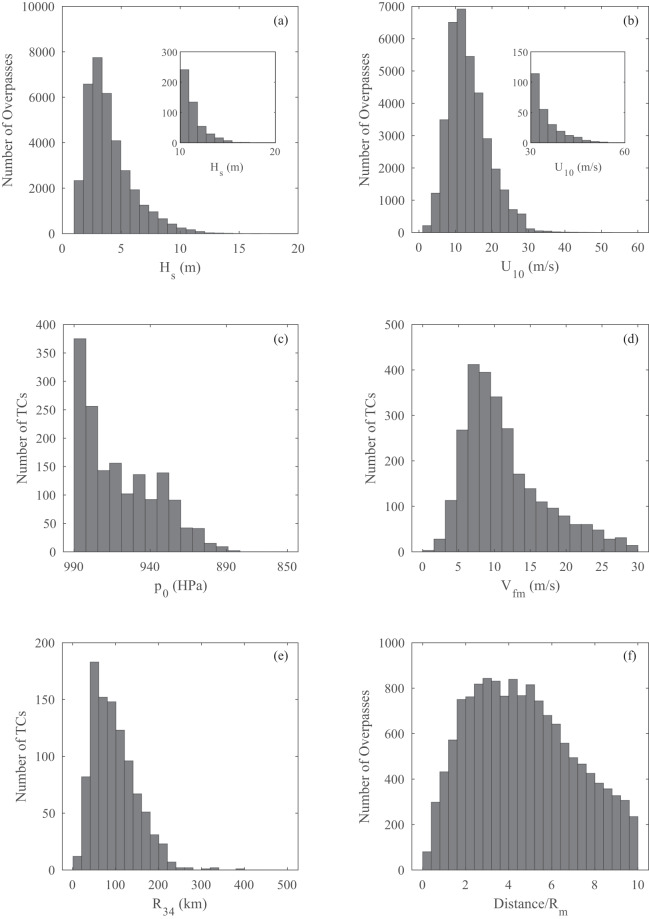
Summary of the altimeter TC database: (**a**) maximum significant wave height *H*_*s*_ for each pass of an altimeter, (**b**) maximum wind speed *U*_10_ for each pass of an altimeter, (**c**) central pressure *p*_0_ of each storm in the database, (**d**) velocity of forward movement *V*_*fm*_ of each storm in the database, (**e**) radius to gales *R*_34_ of each storm in the database, and (**f**) minimum distance between altimeter and storm eye for each altimeter pass.

The corresponding distributions of observed wind and wave data from the altimeter observations are shown in Fig. 6. As can be seen, the number of observations in the satellite observations is far larger than for buoy data. This is due to the high along-track spatial coverage of the altimeter, together with the fact that it is a global dataset compared to the buoy data being confined to North America. The full dataset contains more than 36,000 altimeter passes over TC wind/wave fields from more than 2,730 TCs. The distributions of the various parameters are similar to the buoy dataset. However, it is noticeable that the maximum recorded values of *H*_*s*_ ≈ 16 m and *U*_10_ ≈ 50 ms^−1^. These values are both lower than the corresponding values from the buoy observations, despite the fact that the altimeter dataset contains many more observations from a larger set of TCs. This suggests that the altimeter database misses some of the extreme observations within the storms. This assumption is supported by comparisons of the distributions of the values of observation distance/*R*_*m*_. For the altimeter dataset, there are few observations near the centres of the TCs. This is because the QA process rejected many observations where the quality of the altimeter returns had been degraded by high rain rates near the centres of storms.

The corresponding distributions of wind speed from the scatterometer observations are not shown here due to space limitations but are similar to those shown in Figs. 5 and 6. This dataset consists of more than 13,500 scatterometer passes through more than 800 TCs. Due to the broad ground track swaths of scatterometers, there are more than 14,000,000 observations.

## Data Records

The dataset is stored as NetCDF files, with one file per TC (a total of 2927 files). The file names largely follows the IBTrACS naming convention, such that it acts as a TC identifier. The general format is: yyyydddHaabbb_TCName.nc, where

- yyyy- Year of occurrence of TC
- ddd- day number from start of year to first observation
- H- N or S for Northern or Southern Hemisphere
- aa- latitude of first observation (no sign)
- bbb- longitude (east) of first observation
- TCName- Name of TC, if this has been allocated

As an example, “2005236N23285_KATRINA.nc” contains the data for Hurricane Katrina with the data commencing on day 236 of year 2005 at latitude 23°N and longitude 285°E. The data stored in each file is described in Tables 1, 2 and 3, with Hurricane Katrina used as an example.

**Table 1:** Dimensions of variables in NetCDF file.

Dimensions	Description	Katrina example
Point	Number of IBTrACS track locations where position, time and TC parameters are given	34
R34_dimension	Number of values of radius to gales at each track location – there can be a maximum of four such values, one for each quadrant	4
Altn	Maximum number of observation points for any of the altimeter passes	165
Altcolumn	Number of columns of data for altimeters	6
Radn	Maximum number of observation points for any of the radiometer passes	2619
Radcolumn	Number of columns of data for radiometers	5
Scattn	Maximum number of observation points for any of the scatterometers passes	2960
Scattcolumn	Number of columns of data for scatterometers	6
MET_Buoy_n	Maximum number of observations times for data from the “MET_Buoys”.	115
MET_Buoy_column	Number of columns of data for the “MET_Buoys”.	3
MET_Buoys_List	Number of buoys measuring meteorological data	25
Dir_Buoy_n	Maximum number of observations times for data from the “DIR_Buoys”.	115
Dir_Buoys_List	Number of buoys measuring directional wave data	16
Frequency_t1	Number of frequencies in wave spectrum (definition 1)	47
Frequency_t2	Number of frequencies in wave spectrum (definition 2)	38

Example shown for Hurricane Katrina.

**Table 2:** Variables related to IBTrACS and satellite data in NetCDF files.

Variable Name	Dimension	Katrina example	Comment
**IBTrACS params**.			
Storm_Time	Point	34	Julian days from January 0, 0000
Storm_Latitude	Point	34	Latitude in deg. north
Storm_Longitude	Point	34	Longitude in deg. east
Storm_P0	Point	34	TC central pressure, HPa
Storm_Pn	Point	34	Atmospheric pressure far from TC, HPa
Storm_Rm	Point	34	TC radius to max. winds, km
Storm_R34	Point,R34_dimension	34,4	TC radii to gales
**Satellite params**.			
Alt_obs	Altn,Altcolumn,Point	165,6,34	For each altimeter observation there are 6 parameters, the altimeter pass will appear associated with the TC location at the closest time to the pass (third dimension). All other times have -999 fill param.
Rad_obs	Radn,Radcolumn,Point	2619,5,34	As for altimeter
Scatt_obs	Scattn,Scattcolumn,Point	2960,6,34	As for altimeter

Example shown for Hurricane Katrina.

**Table 3:** Variables related to buoy data in NetCDF files.

Variable Name	Dimension	Katrina example	Comment
**Non-Dir Buoy obs**.			
MET_Buoys_ID	MET_Buoys_List	25	NDBC buoy ID number
MET_Buoys_Latitude	MET_Buoys_List	25	Lat. of buoys, deg. N
MET_Buoys_Longitude	MET_Buoys_List	25	Long. of buoys, deg. E
MET_Buoys_obs	MET_Buoy_n, MET_Buoy_column, MET_Buoys_List	115,3,25	Three values (time, Hs, U10) defined by col. 2. Data values for MET_Buoys_List in column three. Fill data -999.
**Dir Buoy obs**.			
Frequency_type1	Frequency_t1	47	No. of spectral freq., Hz
Frequency_type2	Frequency_t2	38	No. of spectral freq., Hz
Dir_Buoys_ID	Dir_Buoys_List	16	NDBC buoy ID number
Dir_Buoys_Latitude	Dir_Buoys_List	16	Lat. of buoys, Deg. N
Dir_Buoys_Longitude	Dir_Buoys_List	16	Long. Of buoys, Deg. E
Dir_Buoys_Time	Dir_Buoys_n,Dir_Buoys_List	115,16	Time in Julian days of each observations of a spectrum.
Dir_Buoys_Spectral_Wave_Density	Dir_Buoy_n, Frequency_t1, Dir_Buoys_List	115,47,16	1D spectrum at each buoy and each time (m^2^s). For data with 38 frequency bands, additional array elements filled with −999.
Dir_Buoys_Mean_Wave_Dir	Dir_Buoy_n, Frequency_ty1, Dir_Buoys_List	115,47,16	Mean dir. for each freq., time and buoy. Deg.
Dir_Buoys_Wave_Spectrum_r1	Dir_Buoy_n, Frequency_t1, Dir_Buoys_List	115,47,16	First normalized polar moment –${r}_{1}^{2}={a}_{1}^{2}+{b}_{1}^{2}$
Dir_Buoys_Principal_Wave_Dir	Dir_Buoy_n, Frequency_t1, Dir_Buoys_List	115,47,16	Princp. dir. for each freq., time and buoy. Deg.
Dir_Buoys_Wave_Spectrum_r2	Dir_Buoy_n, Frequency_t1, Dir_Buoys_List	115,47,16	Second normalized polar moment –${r}_{2}^{2}={a}_{2}^{2}+{b}_{2}^{2}$

Example shown for Hurricane Katrina.

The dataset^56^ can be downloaded from 10.26188/24471688.

To provide uses an example of how to access and use the NetCDF files, the Matlab script used to produce Fig. 4 can be downloaded from 10.26188/24903117.

Tables 1, 2 and 3 below contain details of the Variable names, definitions of the measured quantities and the dimensions of the storage array within the NetCDF files of the TC database.

## Technical Validation

The instruments used to compile the present database are all extensively used for metocean applications, however, the extreme conditions in TCs pose issues for all instrumentation systems. Below, calibration and validation studies of these systems under TC conditions are considered.

### Buoys

Surface floating buoys have a history of more than 50 years of usage and form the basis for national wave measuring programs around the world. These systems use either acceleration or GPS principles to measure the time series of surface displacement (Note that NDBC data use acceleration). These systems have been extensively calibrated and validated and represent mature technology.

Anemometers (both cup and sonic) mounted on meteorological buoys also provide the mainstay of metocean wind measurements. Again, at high winds and large sea states, concerns have been raised about tilting of the buoy axis and shadowing by large waves^11,22–24^.

### Altimeters

The altimeter wind speed and wave height data used in the database are obtained from the AODN archive^45,55^. The altimeter data in the archive were calibrated and validated against extensive buoy data^45^. These data indicate no decrease in the accuracy of measurements of significant wave height up to 10 m. Beyond this value, there is almost not co-located data to make an assessment. Similar calibration of wind speed against buoy data are used for wind speeds up to approximately 25 ms^−1^. For higher wind speeds, data suggests that the radar cross-section of the altimeter signal is less sensitive to wind speed and a correction to these calibrations is applied at higher wind speeds^57^. These altimeter calibration relations for wind speed have been subsequently validated against scatterometer and radiometer data^58^.

### Scatterometer

The scatterometer data used in the present database were also sourced from the AODN archive. The scatterometer values of wind speed were calibrated and validated against global datasets^50^. Ribal *et al*.^54^ subsequently proposed a high wind speed correction for TC conditions. The present dataset does not apply this high wind speed correction. However, it is a very simple process to correct the present data before application and this is recommended to users.

## Data Availability

The data were extracted from each of public archived and searched to extract data associated with the TC using Matlab codes. The NetCDF files were written using Matlab codes. These codes are available at 10.26188/24515113^59^.
